# Presence-absence surveys yield spatially imprecise information about nesting sites of an endangered, forest-nesting seabird

**DOI:** 10.1371/journal.pone.0315531

**Published:** 2024-12-12

**Authors:** Jonathon J. Valente, Lindsay J. Adrean, S. Kim Nelson, Matthew G. Betts, Daniel D. Roby, James W. Rivers

**Affiliations:** 1 U.S. Geological Survey, Alabama Cooperative Fish and Wildlife Research Unit, College of Forestry, Wildlife and Environment, Auburn University, Auburn, AL, United States of America; 2 American Bird Conservancy, The Plains, Virginia, United States of America; 3 Department of Fisheries, Wildlife, and Conservation Sciences, Oregon State University, Corvallis, Oregon, United States of America; 4 Department of Forest Ecosystems and Society, Oregon State University, Corvallis, Oregon, United States of America; 5 Department of Forest Engineering, Resources, and Management, Oregon State University, Corvallis, Oregon, United States of America; MARE – Marine and Environmental Sciences Centre, PORTUGAL

## Abstract

Presence-absence surveys are frequently used to monitor populations of rare and elusive species. Such data may also be used as a proxy for breeding activity, but links between presence-absence data and higher-order processes must be validated to determine their reliability. The Marbled Murrelet (*Brachyramphus marmoratus*) is a threatened seabird that nests in older-aged forests along the Pacific Coast. Its nests are exceptionally difficult to find, so we tested whether presence-absence surveys can help identify nesting sites. Between 2018 and 2022 we located 17 trees containing active murrelet nests in the Oregon Coast Range (USA) and 38 trees that purportedly contained no active nests (26 in occupied murrelet stands and 12 in unoccupied stands). Observers surveyed within 200 m of focal trees using standard presence-absence surveys, and we modeled the effects of site status (active nest or control) and distance from the focal tree on probability of recording murrelets. We never detected murrelets in unoccupied control sites. We found some evidence that the probability of recording presence was higher at active nesting sites (0.81, 95% CI: 0.71, 0.88) than at occupied control sites (0.71, 95% CI: 0.64, 0.78) although a null model had similar support. The probability of recording murrelet breeding behaviors in nesting and occupied control sites was 0.20 (95% CI: 0.14, 0.27) regardless of distance to a known active nest. These results suggest that presence-absence surveys may be useful for identifying plausible murrelet nesting habitat, but they are ineffective for identifying active nesting sites. Moreover, we estimated that 20 repeated surveys at a point in space are required to reasonably conclude there are no active nesting sites within 200 m. These findings serve as an important reminder of the limitations that can come with relying on presence-absence data alone to identify breeding sites.

## Introduction

Effective monitoring of population change is necessary to understand and mitigate anthropogenic biodiversity losses [1–4]. For rare and declining species, identifying spatial distributions can be complicated by sampling inefficiencies associated with detectability of small populations and cryptic behavior of target species. In many cases, presence-absence (i.e., detection-nondetection) data are used as an alternative to monitoring abundance because of cost-effectiveness, the ease of implementation at large scales, and the feasibility of data collection methods [5, 6]. Additionally, presence-absence surveys can be preferable to more intensive and invasive sampling methods (e.g., capturing and marking) that may alter behavior and fitness outcomes [7]. Fortunately, improved modeling techniques over the last several decades have enhanced our ability to generate unbiased estimates of occupancy in the presence of imperfect detection [5, 8–10], increasing the appeal of presence-absence surveys for researchers.

Despite the many advantages of presence-absence sampling, understanding what biological inferences can be drawn from such data remains an important challenge. For example, identifying and protecting breeding habitat is critical for supporting healthy populations, yet in many cases it is ambiguous whether presence of a species can be a reliable indicator of its breeding activity. Theory predicts that the probability an individual occupies a site should increase with habitat quality [11], which has been supported by empirical evidence in many species, including birds [12–14], mammals [15, 16], and insects [17, 18]. Yet presence may not always indicate habitat quality [19]. Mobile species, for example, may be encountered while commuting to breeding sites or while prospecting in sites that are ultimately not selected for breeding [20, 21]. Furthermore, mismatches between factors driving habitat selection and those affecting fitness can result in breeding attempts in poor quality habitat [22]. Indeed, spatially explicit simulations have demonstrated that reproductive success is generally a weak predictor of species presence [23]. Thus, the relationship between presence and breeding activity can be complex and idiosyncratic.

The Marbled Murrelet (*Brachyramphus marmoratus*; hereafter, murrelet) is a seabird that employs a “habitat split strategy” wherein it forages in nearshore marine areas throughout the year but also nests within late-successional and old-growth coastal forests during the April—August breeding season [24]. Murrelets inhabit coastal areas from Alaska to central California and are considered threatened in all but the northernmost portion of their range [24]. Despite two decades of legal protections, populations have failed to recover [25] due to ongoing loss and fragmentation of old-growth breeding habitat [26] and climate-induced changes in oceanic conditions that reduce food availability and breeding activity [27–30]. Locating active nesting sites of this species is exceptionally difficult because murrelets lay a single egg on platform branches high off the ground and approach their nests at high speeds predominantly during the pre-dawn and dusk hours [24]. Consequently, murrelets have largely been monitored for the past 20 years by surveying populations at sea [25], and much less effort has been devoted towards locating and monitoring active nests [24].

Despite these challenges, delineating murrelet nesting habitat is critical given this species’ endangered status and because its breeding range overlaps with some of the most productive timber forests in the world [31, 32]. In 1990, the Pacific Seabird Group (PSG) first developed [33] and then revised [34, 35] a presence-absence survey protocol to identify forested areas with high likelihood of being used by nesting murrelets (hereafter, the PSG survey). These surveys involve trained observers watching and listening for murrelets in the early morning hours during the breeding season while recording detections and associated behaviors. Sites are classified as “occupied” when observers record murrelets calling from a stationary location, flying below the canopy, or circling above the canopy, behaviors that typically occur around active nesting sites (hereafter, “breeding behaviors”; [36–39]). Thus, occupied sites are assumed to have importance for breeding [34, 35]. At present, however, it is unclear how far from an active nesting site these breeding behaviors occur and thus whether they are helpful for identifying active nesting sites, or merely for delineating potential habitat. Understanding the spatial scale to which these detections are relevant is crucial given that PSG surveys are now the accepted method for identifying murrelet nesting sites on public and private timberlands [35].

In this study, we tested the hypothesis that presence-absence data generated by PSG surveys can be used to identify trees that contain active nesting sites. We used an experimental approach where trained observers who were naïve to murrelet nest locations conducted PSG surveys at variable distances from trees with known, active nesting sites, and at variable distances from randomly selected potential nesting trees. If our hypothesis was supported, we expected the frequency of recording murrelet breeding behaviors to (1) be greater near active murrelet nesting sites than at random potential nesting sites and (2) decrease with distance from an active nesting site. Results from this study will help elucidate the relationship between detected breeding behaviors and true breeding activity for this highly mobile species and help us understand the spatial scale of breeding behaviors in relation to active nesting sites.

## Materials and methods

### Ethics statement

This study was carried out under guidance from the Oregon State University Institutional Animal Care and Use Committee and with appropriate permits from Oregon Department of Fish and Wildlife and the U.S. Fish and Wildlife Service.

### Study area and experimental design

**Fig 1:**
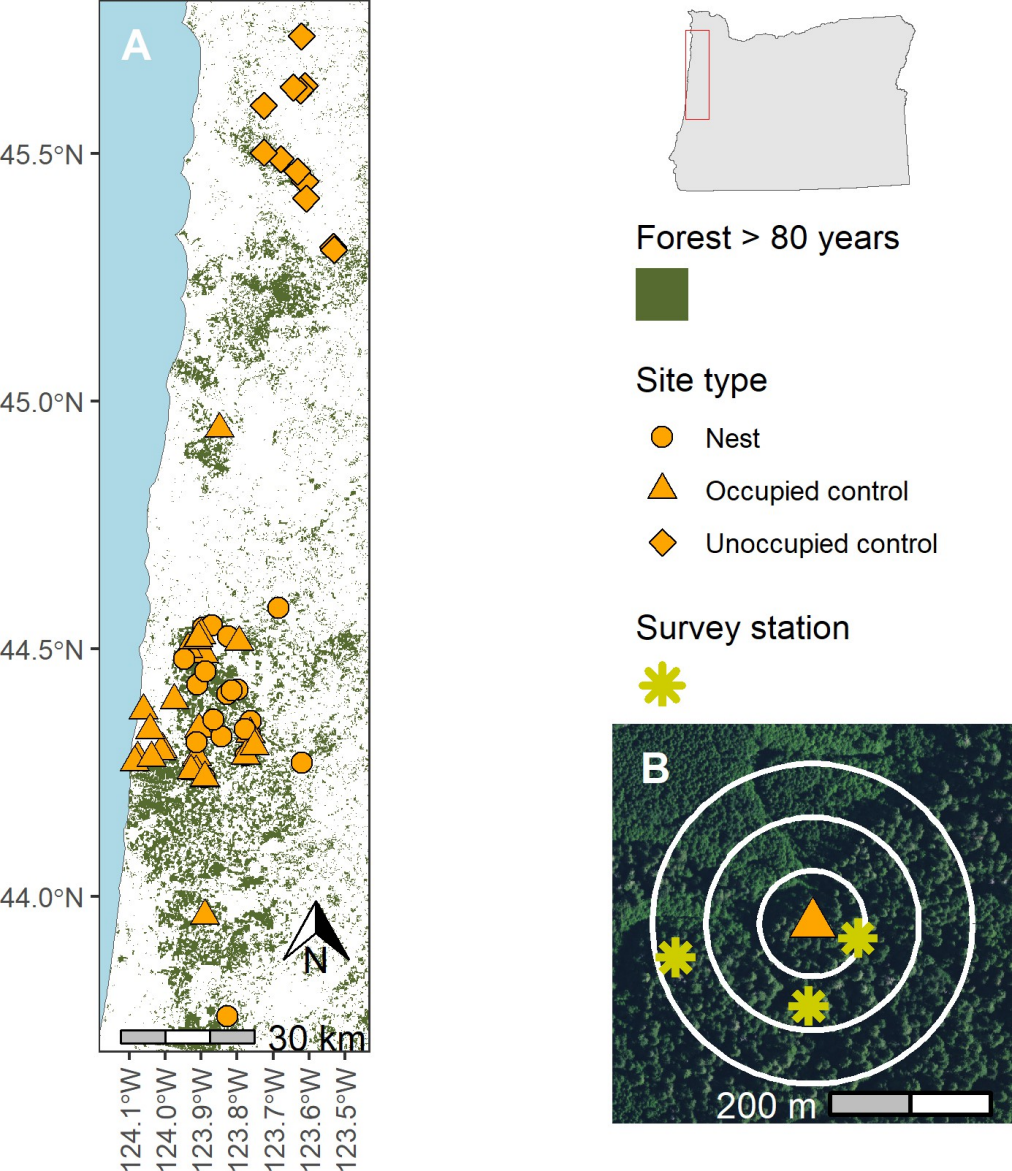
(A) Study area on the central coast of Oregon with locations of nesting sites and focal occupied and unoccupied control sites; (B) Example of a study site depicting the location of an occupied control site with one survey station within each distance bin (white circles; 0–67 m, 67–133 m, and 133–200 m) around the focal tree. Imagery in (B) is publicly available from the 2020 National Agriculture Inventory Program (U.S. Department of Agriculture Farm Production and Conservation—Business Center, Geospatial Enterprise Operations).

We conducted this experiment as part of a larger study on murrelet breeding and movement ecology along the central coast of Oregon, USA, centered on the city of Newport (N 44°38’12.42”, W 123°3’12.43”). In this region, murrelet breeding habitat is found in late successional and old-growth forest of the Oregon Coast Range, across a diversity of ownerships. We collected data over the course of four murrelet breeding seasons, April—August of 2018, 2019, 2021, and 2022. Between April 21 and June 7 each year, we captured 49–76 murrelets (239 total) at sea between Lincoln City, OR and Yachats, OR, fitted a subset of them with VHF radio-tags, and then followed the movements of tagged individuals throughout the breeding season, including tracking those that moved inland to their nests. We initially approximated locations of nesting sites using telemetry receivers in fixed-wing aircraft, and pinpointed exact nest locations with ground-based visual surveys (Fig 1A). Additional details regarding capture and tracking can be found in [30]. Once we located a nest, we installed nest cameras with infrared capabilities in adjacent trees and recorded digital video footage 24 h per day to monitor the presence of murrelets at the nest. Trained observers later reviewed footage from each camera and documented each time an adult visited the nest for activities such as incubation, brooding, or chick-feeding.

Our study used active nesting and control sites; both site types focused on a tree that was surrounded by “stations” where we conducted PSG surveys (Fig 1B). Our 17 nesting sites were centered on trees containing active nests we had identified using telemetry. We identified control sites from a randomized list of forest stands that had been previously surveyed using the PSG protocol (Table 1). In 2018 we chose 12 “occupied control sites” in which breeding behaviors had previously been recorded, and another 12 “unoccupied control sites” where no breeding behaviors had been recorded. In 2019–2022, we selected one occupied control site to pair with each active nesting site we found but stopped sampling unoccupied control sites due to the complete lack of murrelet detections at these sites in 2018. Within control sites we used aerial imagery to randomly select a focal tree that was >25 m from the forest edge and that contained large horizontal branches that could serve as potential murrelet nesting platforms.

**Table 1:** The number of sites sampled using Marbled Murrelet audiovisual surveys between 2018 and 2022 along the central coast of Oregon, USA. Each site had multiple stations within 200 m of focal trees, and each station was surveyed multiple times. During each survey, observers recorded presence if any murrelets were encountered, and occupancy if breeding behaviors were noted.

	Year	Sites	Stations	Surveys	Occupied surveys	Presence surveys
Active nesting sites						
	2018	4	12	42	15 (35.7%)	34 (81.0%)
	2019	2	6	13	4 (30.8%)	13 (100%)
	2021	5	15	44	7 (15.9%)	27 (61.4%)
	2022	6	17	46	12 (26.1%)	38 (82.6%)
	Total	17	50	145	38 (26.2%)	112 (77.2%)
Occupied control sites						
	2018	12	35	131	25 (19.1%)	95 (72.5%)
	2019	2	6	19	3 (15.8%)	13 (68.4%)
	2021	6	16	61	14 (23.0%)	37 (60.7%)
	2022	6	18	48	13 (27.1%)	34 (70.8%)
	Total	26	75	259	45 (17.4%)	179 (69.1%)
Unoccupied control sites						
	2018	12	34	151	0 (0.0%)	0 (0.0%)
	Total	12	34	151	0 (0.0%)	0 (0.0%)

### Sampling

We established survey stations around each focal tree in our study at which we conducted PSG surveys [34]. We placed one station at randomly selected locations in each of three distance bins around the focal tree: 0–67 m, 67–133 m, and 133–200 m (Fig 1B). We chose 200 m as a maximum distance because it coincides with previous observations of murrelets flying below the canopy for nearly 200 m when approaching or departing nesting sites [40]. Observers used random number tables to identify the initial distance and direction from the focal tree for each station, then followed the methods set in the PSG Survey Protocol to ensure stations contained at least a partial view of the sky and were located away from sources of noise (e.g., a loud stream) to maximize murrelet detectability. Per the protocol, observers could move stations up to 50 m from the initial coordinates if necessary. At some sites we were unable to identify 3 locations that were suitable for a sampling station. Therefore, active nesting sites had a mean (SD) of 2.94 (0.24) sampling stations and control sites had a mean of 2.88 (0.43) sampling stations.

We hired independent observers to conduct surveys at our stations. Observers were instructed to survey each station 4–5 times during the breeding season (Table 1), and all observers were naïve as to which stations were close to active nests. All surveys were conducted between 10 May and 25 August, and surveys at nesting sites that occurred after the nest became inactive (as determined from nest video monitoring) were removed from analyses. Table 1 summarizes survey efforts across all site types. Surveys followed the methods established in the PSG inland survey protocol [34], beginning 45 min prior to sunrise and concluding 75 min after sunrise, during which time observers recorded audio and visual murrelet detections. During surveys, observers recorded a murrelet as “present” if the species was detected at all and recorded “occupancy” if they observed murrelet breeding behavior, which included: a murrelet nest, egg, or chick; or an adult landing in a tree, calling from a stationary position, flying below the canopy, or circling above the canopy. In the analyses described below, we analyzed occupancy and presence detections separately.

### Data analysis

We used mixed effects logistic regression to examine the effects of site status (active nest vs. occupied control) and distance from the focal tree on both the probability of recording occupancy and the probability of recording presence during a survey. No murrelet detections were recorded at unoccupied control sites, so we excluded these from formal statistical analysis because 0 is on the boundary of the parameter space and therefore inestimable with logistic regression. For each response variable (occupancy and presence) we built four models to represent the predictions that detection probabilities are (1) not affected by site status or station distance from focal tree (response ~ 1), (2) greater at active nesting sites but do not vary with station distance (response ~ nest), (3) greater at nesting sites and decline linearly with station distance (response ~ nest + distance + nest*distance), and (4) greater at nesting sites and vary non-linearly with station distance (response ~ nest + distance + distance^2^ + nest*distance + nest*distance^2^; Table 2). We included site and station as random effects in all models of occupancy; for presence models we included station as a random effect but excluded site effects to mitigate problems with model convergence. We fit all models in R version 4.3.1 using the lme4 package [41]. For both the occupancy and presence analyses we compared among the 4 models using Akaike’s Information Criterion corrected for small sample size (AICc; [42]). We calculated ΔAICc for each model by subtracting the AICc value for the most parsimonious model from the target model’s AICc value, and we considered any model with a ΔAICc less than 2 to have strong support. We also calculated the AICc weight for each model i as:

$$w_{i}=\frac{exp(-0.5*{\Delta AICc}_{i})}{\sum_{r=1}^{R}exp(-0.5*{\Delta AICc}_{r})}$$

AICc weight is interpreted as the probability that model i is the best of the set of candidate models. Finally, we tested the fit of each model by comparing the chi-square statistic calculated from the empirical data to a distribution of chi-square statistics calculated from 500 datasets parametrically bootstrapped from the fitted model [43]. We considered the model a reasonable fit if it fell within a 95% confidence interval (CI) of the bootstrapped values. Data generated during this study are available as a U.S. Geological Survey data release [44], and analysis code are available via a U.S. Geological Survey software release [45].

**Table 2:** Ranking of the models fit to test whether the presence of an active nest and distance from the nest influenced probability of detecting Marbled Murrelets during presence-absence surveys conducted between 2018 and 2022 along the central coast of Oregon, USA. We used mixed effects logistic regression to model the probability that naive observers recorded murrelet occupancy or presence. For each model we report the small-sample corrected Akaike Information Criterion score (AICc), difference between the model’s AICc and that of the top model (ΔAICc), Akaike weight of evidence (wi), number of model parameters (K), and the log likelihood.

Model		AICc	ΔAICc	w_i_	K	log likelihood
Occupancy						
	~1	431.74	0.00	0.48	3	-212.84
	~nest	433.05	1.31	0.25	4	-212.47
	~nest + distance + distance^2^ + nest*distance + nest*distance^2^	433.69	1.95	0.18	8	-208.66
	~nest + distance + nest*distance	435.09	3.36	0.09	6	-211.44
Presence						
	~nest	473.74	0.00	0.46	3	-233.84
	~1	474.35	0.61	0.34	2	-235.16
	~nest + distance + nest*distance	475.61	1.87	0.18	5	-232.73
	~nest + distance + distance^2^ + nest*distance + nest*distance^2^	479.68	5.94	0.02	7	-232.70

## Results

**Fig 2:**
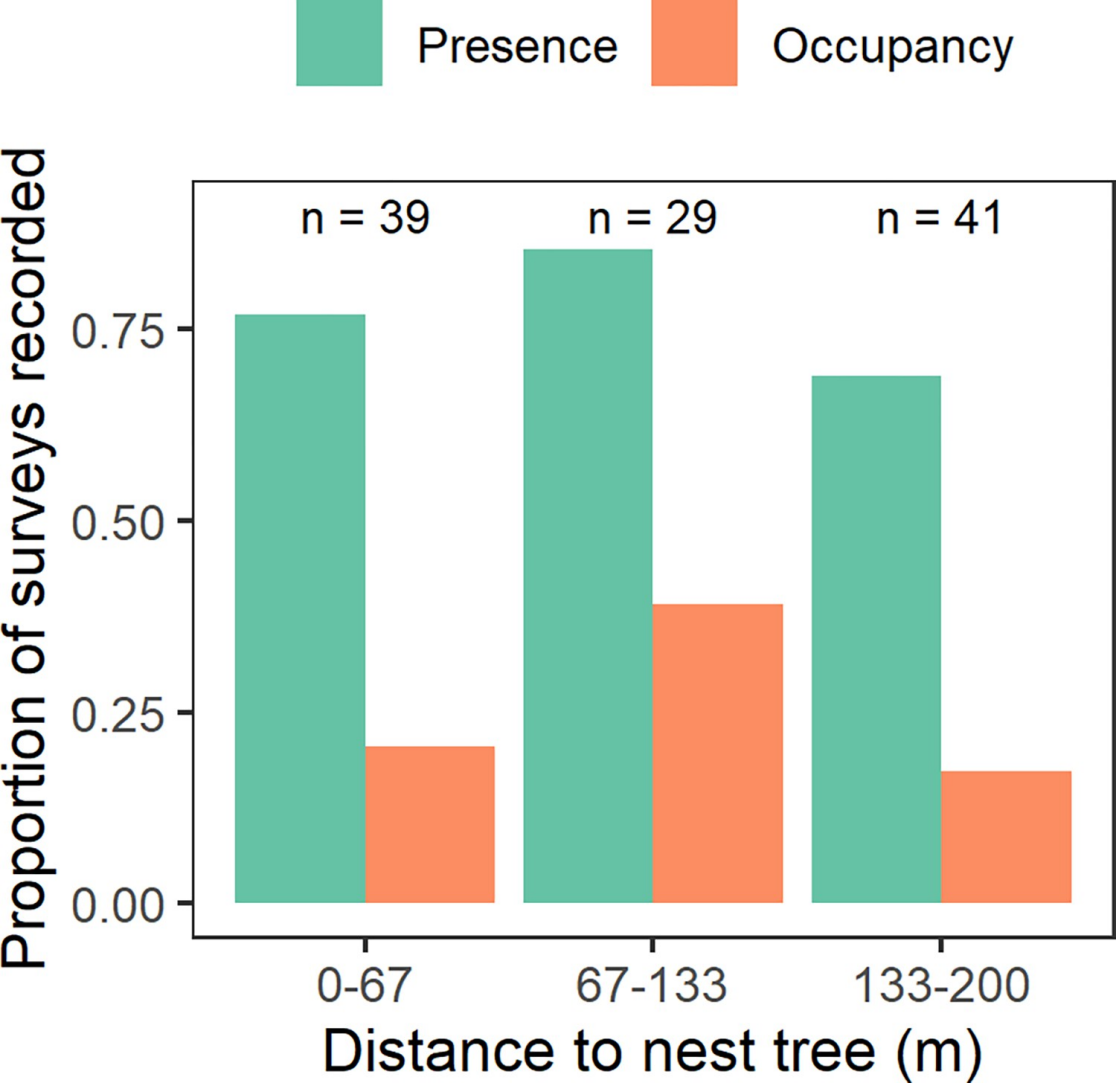
Between 2018 and 2022 observers conducted at least 109 surveys along the central Oregon Coast, USA, during which a murrelet arrived at and/or departed from an active nest. This figure shows the proportion of those surveys during which observers recorded any murrelet activity (presence) or purported breeding behaviors (occupancy).

Both occupancy and presence were recorded more frequently at active nesting sites, less frequently at occupied control sites, and never at unoccupied control sites (Table 1). Breeding behaviors that indicate occupancy were infrequently detected by observers, even when they unequivocally occurred. Using video recordings, we were able to confirm that murrelets arrived at and/or departed from active nesting sites while at least 109 (27%) PSG surveys were being conducted at associated stations. Observers recorded some form of murrelet activity (presence) during 85 (78%) of those confirmed arrival/departure surveys but recorded breeding behaviors (occupancy) during only 29 (27%; Fig 2).

Across all surveys, 98% of breeding behavior (occupancy) detections that were recorded consisted of either sub-canopy flights or above-canopy circling. Observers recorded one occasion of an adult landing in a tree and one occasion of an adult calling from a stationary location; both observations occurred at active nesting sites. We found that the ratio of sub-canopy flight to above-canopy circling detections differed between nesting and occupied control sites (Z = 2.22, p = 0.03). Sub-canopy flights comprised 63.2% of breeding behavior detections at active nesting sites while circling comprised 31.6% of detected breeding behaviors. At occupied control sites, however, sub-canopy flights comprised 90.9% of detected breeding behaviors whereas circling only accounted for 9.1% of detections.

**Fig 3:**
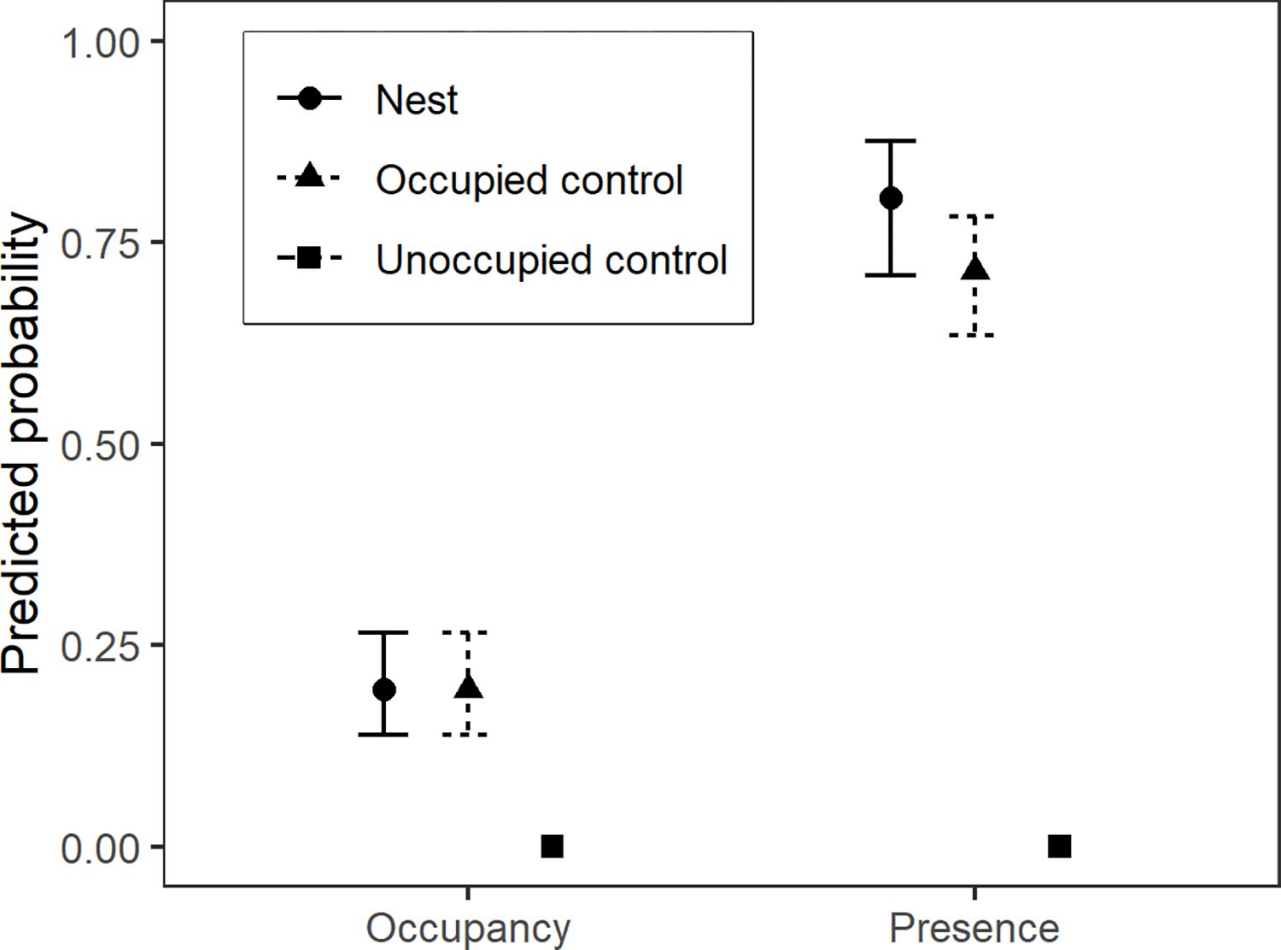
Comparison of the predicted probabilities (95% confidence intervals) of detecting Marbled Murrelet occupancy and presence during Pacific Seabird Group surveys conducted from 2018 to 2022 along the central Oregon Coast, USA. Distance from the survey station to a known active nest is not depicted because top models indicated this did not affect probabilities of recording murrelets.

Within occupied forest stands, we found no strong evidence that proximity to a known active nesting site influenced the probability of recording breeding behaviors during a PSG survey. Based on AICc model comparison, the null model, which excluded both site status (nesting/occupied control) and distance, had the greatest support among competing models (Table 2). Results from this model indicated that the probability of detecting breeding behaviors (occupancy) on any given survey was 0.195 (95% CI: 0.139, 0.266) regardless of the presence of, or proximity to, a known active nest (Fig 3). Two additional models—one containing status only and the second containing status plus its interaction with quadratic distance—also had support, as indicated by ΔAICc values less than 2 (Table 2). However, the summed weight of these two models (0.43) was less than the weight of the null model (0.48), indicating the latter is a more parsimonious representation of the data. Findings from our bootstrapping procedure indicated that the null model was a suitable fit for the data (p = 0.642).

We found weak evidence that the probability of recording presence was greater near known active nesting sites than at occupied control sites. The model with the lowest AICc value estimated that the probability of recording presence during a survey was 0.805 (95% CI: 0.709, 0.875) at sites with a known active nest and 0.714 (95% CI: 0.635, 0.782) at occupied control sites (Fig 3). However, the null model (which excluded both site status and distance effects) also had strong support with a ΔAICc value of 0.61. Indeed, the AICc weight of the null model was 0.34 which indicates it is only slightly less probable than the top model with a weight of 0.46 (Table 2). Thus, although the model that included nest effects is the most plausible representation of the data, the null model cannot be ruled out. Comparatively, there was much less support for the two models that included distance covariates, as their summed AICc weights were only 0.2. Bootstrapping suggested that the top model was a suitable fit for the raw data (p = 0.624).

## Discussion

Detecting breeding behavior for cryptic species can be logistically challenging and financially costly. While presence-absence data tend to be much easier to collect, it is critical to link such detections to a focal species’ biology (e.g., reproductive activity) to understand what information is contained in such detections. In this study, we found that murrelet detections were more common in occupied habitats than in those previously determined to be unoccupied. However, within occupied murrelet habitat, there was no strong evidence that observers were more likely to detect murrelets or murrelet breeding behaviors (i.e., sub-canopy flights or overhead circling), near active nests than at random potential nesting sites. Thus, murrelet observations alone in potential nesting habitat are likely insufficient for identifying fine-scale nesting locations. This also implies that murrelets are using habitat that extends well beyond the immediate vicinity of active nesting sites. To what extent such use is due to nesting vs. non-nesting individuals is unknown.

Although purported breeding behaviors used to determine site occupancy have been noted around active nesting sites, no studies have directly examined whether or how frequently these behaviors are exhibited at non-nesting sites. This is because it is extremely difficult—if not impossible—to verify there are no active nests in a stand. Murrelets typically nest > 12 m off the ground [24], and given the species’ cryptic nature, the only way to confidently verify absence is to climb every potential nest tree and examine each platform branch for breeding activity. This was logistically and financially infeasible in our study (and indeed in most studies); nevertheless, to our knowledge our investigation represents the most comprehensive effort to examine the spatial distribution of these behaviors to date.

Given we could not know the locations of all active nests in our nesting or control sites, one potential explanation for our findings is that available and suitable breeding habitat for murrelets is saturated in our study area, such that even randomly placed points in occupied old-forest habitat fall relatively close to active nests. Indeed, murrelet breeding habitat has been dwindling in recent decades [26], which could concentrate densities of breeding birds into remnant habitat patches. Furthermore, our occupied control sites were selected because purported breeding behaviors had been detected within the broader forest stand in previous years. Given murrelets have high fidelity to nest sites and are suspected to be philopatric across years [24], it is possible that some of our control sites contained active nests. Secondly, there is evidence that murrelets use social attraction to identify prospective breeding habitat [46], which could lead to nesting aggregations in stands containing active nests. Therefore, it is possible that survey stations located further from known active nesting sites were relatively close to a concurrently active nest whose presence was unknown. That said, murrelet breeding propensity in our study region was extremely low due to poor ocean conditions (< 10%; [30]) and habitat saturation seems an unlikely explanation for the pattern we observed.

A more straightforward explanation is that murrelet activity may not be a strong indicator of proximity to an active nesting site at the scale of our surveys, regardless of the behavior exhibited. To be clear, this does not necessarily imply that murrelet detections are ineffective for identifying breeding habitat. Indeed, sub-canopy flight was the most commonly documented behavior and there is no reason for individuals to fly below the canopy unless they are prospecting for or approaching a nesting site. At a minimum, sub-canopy flights suggest that murrelets recognize the stand as potential nesting habitat but could also indicate there is an active nest somewhere in the stand [38, 40]. Most of the other breeding behaviors detected in our study involved observations of above-canopy circling. Like other members of the Alcidae, murrelets are social [24, 46] and above-canopy circling is thought to be a form of social interaction in which groups of birds interact before returning to the ocean [24]. Although this behavior has been observed predominantly above stands with known active nests, non-breeders and possibly individuals nesting in other stands are thought to join such aggregations, especially late in the breeding season [24]. Consequently, the relative frequency of these circling behaviors over active nesting sites compared to random locations remains ambiguous.

Although we found no strong evidence that detections of murrelet breeding behaviors are greater near active nesting sites, it is important to note the limited sample size in our study. Over the course of 4 breeding seasons, we were only able to find 17 active nests despite radio-tagging 239 individuals due to the species’ low breeding propensity [30]. As with any study, a larger sample size would yield greater power to distinguish subtle differences in detection rates between site types. Nonetheless, our methods were an effective and unbiased approach for finding murrelet nests, and our sample size was comparable to similar studies with this species [47–50].

Our findings do, however, provide useful data on the frequency at which false negatives are recorded at sites with known active nests (Fig 2) that could be useful for protocol design. Our trained observers failed to record murrelet breeding behaviors on most surveys, even when they were close to an active nesting site (Figs 2 and 3). Thus, failure to record a murrelet at a survey station (e.g., around our control sites) does not necessarily mean the species is absent. Indeed, because we found that observers will fail to detect murrelet occupancy 80% of the time, we estimate that up to 20 repeated surveys at a station near an active nest are needed to ensure that the probability of failing to record murrelet breeding behaviors (occupancy) drops below 0.05 (i.e., 0.86^20^ = 0.049). Importantly, our samples of unoccupied control stands indicated that trained observers never falsely recorded murrelet occupancy during 151 surveys, suggesting that false positives during surveys are rare.

Ultimately, to achieve a more statistically definitive answer to whether occupancy can inform managers of the murrelet nesting status in a patch of habitat, a study would need to identify the locations of all active murrelet nests in an area so researchers have exact distances from surveys to those nests. Thus, until substantial resources are available to more closely examine the relationship between active nesting sites and detectable murrelet activity within a stand, it is prudent to continue using breeding behaviors as an indication of likely nesting. Given that murrelet habitat has declined by nearly 20% [26] since 1988, and that murrelet populations have failed to recover in recent decades [25], it seems advisable to consider occupied stands are used for nesting until data indicating the contrary are obtained.

Our results indicated that detecting breeding behaviors (occupancy) was not a reliable indicator of a nearby active murrelet nesting at a fine-scale, reiterating that researchers should take care when using presence-absence data as a proxy for more complex biological phenomena without a clear link. Although previous research has demonstrated a relationship between occupancy and higher-order variables like abundance [51, 52] or fitness [12–14], such relationships are unlikely to hold for all species. Furthermore, presence can be a misleading indicator of important breeding habitat when factors influencing habitat selection are decoupled from those that improve fitness [19]. Developing a better understanding of what information is contained within presence-absence data will help researchers and conservation practitioners make informed decisions about how the information can be used to support target species.
